# Position, Orientation and Velocity Detection of Unmanned Underwater Vehicles (UUVs) Using an Optical Detector Array

**DOI:** 10.3390/s17081741

**Published:** 2017-07-29

**Authors:** Firat Eren, Shachak Pe’eri, May-Win Thein, Yuri Rzhanov, Barbaros Celikkol, M. Robinson Swift

**Affiliations:** 1 Center for Coastal and Ocean Mapping, University of New Hampshire, 24 Colovos Road, Durham, NH 03824, USA; yuri.rzhanov@unh.edu; 2 National Oceanic and Atmospheric Administration (NOAA), 1315 East West Highway, Silver Spring, MD 20910, USA; shachak.peeri@noaa.gov; 3 Department of Mechanical Engineering, University of New Hampshire, 33 Academic Way, Durham, NH 03824, USA; may-win.thein@unh.edu (M.-W.T.); barbaros.celikkol@unh.edu (B.C.); mrswift@unh.edu (M.R.S.)

**Keywords:** optical detector array, Unmanned Underwater Vehicles, optical communication, pose detection

## Abstract

This paper presents a proof-of-concept optical detector array sensor system to be used in Unmanned Underwater Vehicle (UUV) navigation. The performance of the developed optical detector array was evaluated for its capability to estimate the position, orientation and forward velocity of UUVs with respect to a light source fixed in underwater. The evaluations were conducted through Monte Carlo simulations and empirical tests under a variety of motion configurations. Monte Carlo simulations also evaluated the system total propagated uncertainty (TPU) by taking into account variations in the water column turbidity, temperature and hardware noise that may degrade the system performance. Empirical tests were conducted to estimate UUV position and velocity during its navigation to a light beacon. Monte Carlo simulation and empirical results support the use of the detector array system for optics based position feedback for UUV positioning applications.

## 1. Introduction

**Figure 1:**
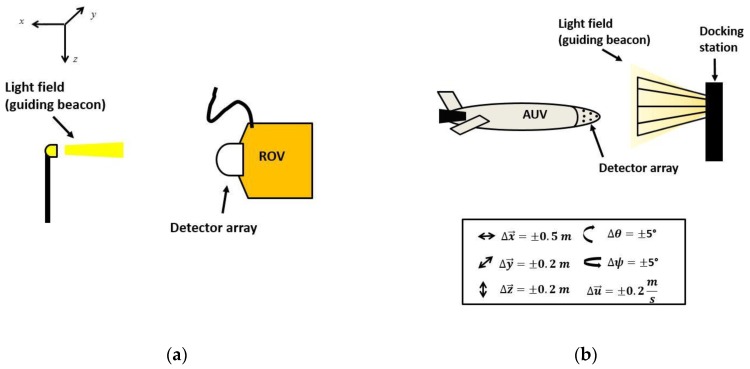
Unmanned Underwater Vehicle (UUV) positioning examples: (**a**) Remotely Operated Vehicle (ROV) dynamic positioning with respect to a light source; and (**b**) Autonomous Underwater Vehicle (AUV) docking station. The light field acts as a guiding beacon with the axis conventions and position and orientation detection parameters used in this study.

Unmanned Underwater Vehicles (UUVs) provide an operational platform for long periods of deployment (on the order of hours) and in depths that are too dangerous for divers [1,2,3,4,5]. UUVs are typically categorized into two groups: (1) Remotely Operated Vehicles (ROVs), which are tethered to the surface ship and manually operated; and (2) Autonomous Underwater Vehicles (AUVs), which are autonomous and provide more flexibility but at the expense of shorter battery life. For both types of UUVs, accurate position and orientation (i.e., pose) detection during navigation is critical for mission operations (e.g., pipeline inspection, surveying and dynamic positioning) where the UUV is required to maintain its pose with respect to a given object of interest [6,7]. One such example is data muling, applications in which a hover capable UUV maintains its pose with respect to an optical transmitter and conducts data transfer [8] (Figure 1a). Accurate positioning is also critical for AUV docking into a physical structure (typically cone type design) to recharge batteries and for data transfer [9] (Figure 1b). Accurate AUV position feedback via its own on-board sensor detection system would help enable successful docking. However, one important challenge for accurate underwater positioning is the communication between platforms, as the radio signals that provide high measurement precision above the water surface do not work efficiently underwater [10].

Currently, most UUVs navigate by using acoustic communication [11,12,13,14,15], as acoustic communication allows for long range of operation (several kilometers) and are commercially available. However, in areas with large traffic volume, such as harbor and recreational fishing areas, the marine environment is acoustically noisy and reduces the performance of the acoustic communication. Recent studies have demonstrated the potential use of both acoustic and optical communication for AUV docking [16,17,18,19]. In these systems, acoustic communication is used for navigation towards the docking station (at ranges longer than 10 m). At shorter ranges (within 10 m), video imagery is used to guide the vehicle into the docking station. In addition, several studies have investigated the use of video imagery with vision-based algorithms for UUV positioning applications [20,21,22]. A system consisting of an optical detector array for underwater free-space optical communication is demonstrated in [23]. However, the system performance has not yet been evaluated for UUV positioning applications.

This paper presents an optical detector array detector system developed to detect UUV pose during its navigation. The specific goal is to develop a cost-effective prototype sensor system that provides position feedback for applications that require precise UUV positioning (e.g., for UUV docking and station keeping applications) [24]. The design used in this study includes a prototype hemispherical photodetector array (5 × 5 array) mounted on a UUV platform as the detector unit and a fixed, single light source with Gaussian beam pattern as a beacon. The performance of the detector array is evaluated based on its capability to detect UUV pose in two different approaches through simulation and empirical measurements. In the first approach, Monte Carlo simulations are conducted to evaluate UUV pose detection during surge, pitch and yaw motions with respect to the light source. (Roll motion detection is not considered due to the use of a single light source with Gaussian beam pattern which is incapable of roll detection.) The impact of varying environmental parameters (i.e., changing water column turbidity and temperature) and hardware noise on the pose detection performance is also evaluated in the Monte Carlo simulations through Total Propagated Uncertainty (TPU) calculations. In the second approach, empirical measurements are conducted to detect UUV translational pose (i.e., surge, sway, and heave) and surge rate as the UUV platform dynamically approaches a fixed light source in a straight-line trajectory. The error tolerance requirements for this study was based on UUV hovering and docking applications. Therefore, the error tolerances were kept within ±0.5 m horizontally in *x*-axis (parallel to the seafloor), ±0.2 m in *y*- and *z*-axis, and ±5° in pitch and yaw [7,9,25]. In addition, the typical entrance velocity of an AUV to a docking station is up to 0.5 m/s with an error tolerance of ±0.2 m/s [12,26]. The empirical tests are conducted at the University of New Hampshire’s Chase Ocean Engineering facilities.

## 2. Materials and Methods

### 2.1. Hardware Design and the Optical Detector Array Output

**Figure 2:**
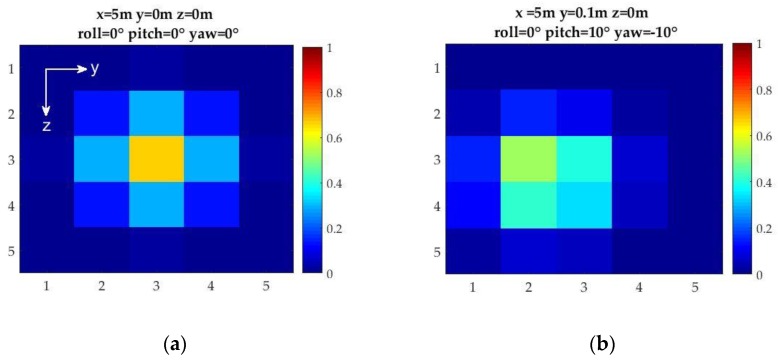
Sample simulator outputs for different relative geometries between the light source and the light detector array with pixel intensities normalized at maximum intensity obtained at *x* = 4 m: (**a**) alignment with *x* = 5 m offset; and (**b**) translation (along the *y*-axis) and rotational offset (pitch and yaw).

The design of the detector array is developed from optical detector array simulator results that the authors obtained via the evaluation of different array geometries based on their capability to generate a unique pose feedback to the UUV [27,28]. The simulator results showed that a hemispherical frame design with a 5 × 5 photo-detector array is sufficient to generate the desired position and orientation feedback to the UUV with a detection accuracy of 0.2 m in translation (surge, sway, and heave) and 10° in orientation (pitch and yaw) based on a Spectral Angle Mapper algorithm (Figure 2). The hemispherical design offered a larger field-of-view than a planar array design and provided more accurate position detection capability. Analytical position and orientation detection algorithms were also developed through static calibrations based on a single light beacon and a detector array mounted on the bow of the UUV [29]. The light source used in the simulations was modeled as a point source Gaussian beam with peak intensity at the origin (i.e., at *x* = 0, *y* = 0, *z* = 0).

**Figure 3:**
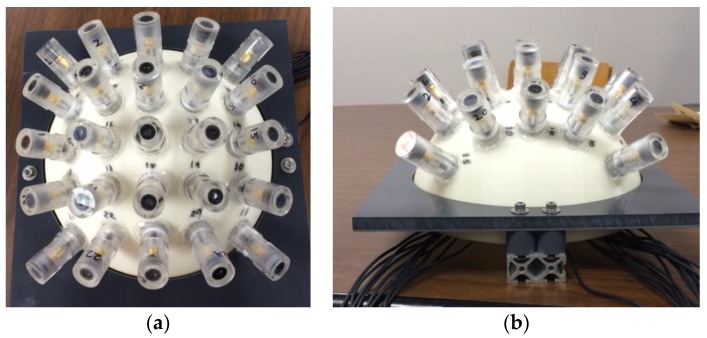
The optical detector array system prototype: (**a**) top view; and (**b**) side view.

The prototype optical detector array design developed in this study includes the following hardware components: twenty-five photodiodes (Thorlabs SM05PD1A) that form the 5 × 5 array, two Analog to Digital converter (ADC) boards, an on-board computer (OBC) with 1 GHz ARM Cortex processor, a power supply (5 V) and reverse-bias circuitry to increase the optical dynamic range. Each photodiode is placed in a waterproof acrylic fixture and the fixtures are concentric to the hemisphere center (Figure 3). The photodiodes sample intensity readings at 5 Hz with 10-bit resolution (0–1023 dynamic range). The spacing between the photodiodes is kept at 0.06 m to eliminate potential cross-talk between the optical detectors [30]. Empirical measurements verified that there was no cross-talk observed in the proposed system.

An Eye Clean-Ace 400 W metal halide light source with T17 bulb is used as a guiding beacon in this study. The light source is operated with an M59 ballast which provides constant power for stable operation. The color temperature for the light source is 6500 K with a Color Rendering Index (CRI) value of 90.

Image moment invariant algorithm is used to convert the optical input (i.e., signature images) sampled on the detector array into pose feedback (the reader is referred to [31,32] for more details). The image moment invariant algorithm is given as:

(1) $$M_{pq}=\frac{1}{S}\underset{i,j}{\sum }{\left(y_{i}-y_{o}\right)}^{p}{\left(z_{j}-z_{o}\right)}^{q}I_{i,j}$$

where $M_{pq}$ is the moment value of order *p* and *q*. *S* is the summation of intensities $\left(I_{i,j}\right)$ and is calculated such that $S=\underset{i,j}{\sum }I_{i,j}$. $y_{i}$ and $z_{j}$ are the row and column coordinates, respectively, of the array elements. The parameters $y_{o}$ and $z_{o}$ denote the coordinates of the pixel with the maximum intensity in the signature image. That is, $P_{max}=\left(y_{o},z_{o}\right)$ is determined with sub-pixel accuracy to provide more accurate calculations of the moment invariants. This results in higher positioning resolution compared to the Spectral Angle Mapper algorithm used in [28]. Image moment invariants are determined up to second order (*p* = 0, 1, 2 and *q* = 0, 1, 2) for efficient real-time calculation without sacrificing significant accuracy. The output of the image moment invariants algorithm is a 3 × 3 matrix, where each element in the matrix provides unique values corresponding to relative pose between the light source and the light detector array.

### 2.2. Calibration Procedures and the Pose Detection Algorithm

Two types of calibration procedures are conducted to accurately determine the UUV pose relative to a light beacon. The first calibration procedure is the geometrical calibration conducted in controlled water clarity conditions. In this calibration scheme, the relative pose between the optical detector array and the light source is varied, and the pose detection algorithms are developed accordingly. The second calibration procedure identifies hardware and environmental parameters that contributes to the TPU of the pose estimations. These parameters include: photodiode response to temperature variations in ambient water conditions, hardware noise and system cross-talk. The underwater light field includes turbidity caused by suspended matter (sediment and biological) carried by currents or turbulence. As a first-order approximation, the simulations are conducted under uniform light field conditions.

#### 2.2.1. Geometrical Calibration and the Pose Detection Algorithm

The geometrical calibration procedure include translational motion along the *x*-, *y*- and *z*-axis and orientation (i.e., pitch and yaw). The experimental translational calibration is conducted in a well-controlled environment at University of New Hampshire (UNH) Jere A. Chase Ocean Engineering facilities. The detector array is immersed underwater in a wave/tow tank with dimensions of 2.44 m in depth, 3.6 m in width, and 36 m in length. The tank is outfitted with a cable-driven tow carriage with actuation that extends through the length of the tank and that can move up to 2.0 m/s. Water clarity conditions in the tank are assumed to be constant with measured diffuse attenuation coefficient, $K_{D}$, of 0.09 $m^{-1}$ [27].

**Figure 4:**
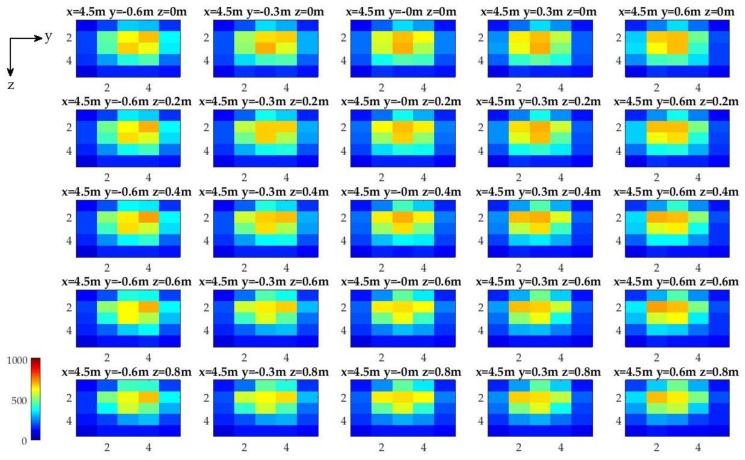
Sample underwater experimental calibration images taken at 4.5 m away from the light source in the *x*-direction. The intensity images (represented by a 10-bit dynamic range (0–1023)) represent the intersection of optical detector array system with the light field from the guiding beacon at different *y*- and *z*-axis offsets.

Translational offsets between the optical detector array and the light source are varied from 4.5 m to 8.5 m at 1 m increments along the *x*-axis, from −0.6 m to 0.6 m at 0.3 m increments along *y*-axis, and from 0 m to 0.8 m at 0.2 m increments along *z*-axis. The light source is located at the origin (i.e., *x* = 0 m, *y* = 0 m, and *z* = 0 m). A total of 125 different position configurations are used for calibration with 200 samples collected at each configuration (Figure 4). The photodiode intensity variations for all calibration measurements are approximately 1% of the maximum photodiode intensity. The image intensities are not perfectly symmetrical about the *y*- and *z*-axis (i.e., with reference to *y* = 0 m and *z* = 0 m). A possible explanation is potential geometrical misalignments in the experimental setup and non-uniform water conditions that may have caused sub-pixel (i.e., between two detectors) shifts of the light field with respect to the detector array.

For the Monte Carlo simulations, a separate calibration procedure is conducted in *x*-, *y*- and *z*-axis; pitch; and yaw directions/motions using the developed simulator [27]. The experimental translational geometry is repeated for the simulated translation calibrations. One percent of the maximum intensity is added as mean noise level to the images to simulate empirical conditions. Pitch and yaw offset quantification is conducted in a similar manner to that of the translational quantification. The relative pitch and yaw between the light source and the detector array is changed in 1° increments from −15° to 15°.

**Figure 5:**
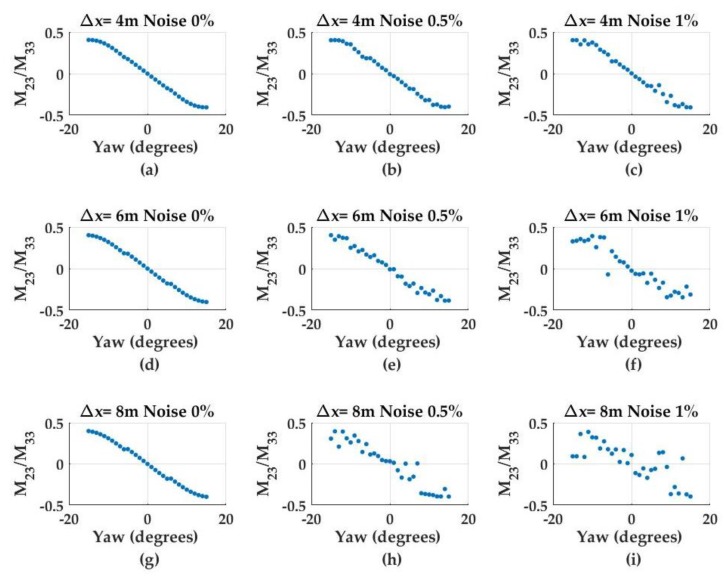
$\frac{M_{23}}{M_{33}}$ ratio values for changing yaw angles at varying *x*-axis offsets (4 m, 6 m, and 8 m) and noise levels (0%, 0.5%, and 1.0%) and with *y*- and *z*-axis offsets at zero: (**a**) Δx = 4 m, noise = 0%; (**b**) Δx = 4 m, noise = 0.5%; (**c**) Δx = 4 m, noise = 1%; (**d**) Δx = 6 m, noise = 0%; (**e**) Δx = 6 m, noise = 0.5%; (**f**) Δx = 6 m, noise = 1%; (**g**) Δx = 8 m, noise = 0%; (**h**) Δx = 8 m, noise = 0.5%; and (**i**) Δx = 8 m, noise = 1%.

UUV *x*-axis position is calculated from the maximum photodiode intensity value, $PD_{max}$, on the array. Because light energy decays exponentially in the water column, as described by the Beer’s law [28], an exponential fit is made to the measurements such that:

(2) $$PD_{max}=a_{1}e^{-b_{1}x_{est}}$$

(3) $$x_{est}=\frac{-\log\left(PD_{max}/a1\right)}{b1}$$

where $PD_{max}$ denotes the maximum photodiode intensity, $a_{1}$ and $b_{1}$ are coefficients obtained from the calibration procedure and $x_{est}$ is the estimated *x*-axis offset between the light source and the detector. UUV pitch and yaw angles are calculated from the 3 × 3 image moment invariant matrix using the ratio of matrix indices. Specifically, the $\frac{M_{32}}{M_{33}}$ ratio is found to correlate with pitch offset (not shown here), whereas the $\frac{M_{23}}{M_{33}}$ ratio correlates with the yaw offset and, as expected, degrades with offset distance and increasing noise (Figure 5).

The analysis shows that there is a linear relationship between the moment index ratios and the pitch and yaw angles. This ratio is also invariant to the changing *x*-axis (i.e., decreasing light energy) as well as the *y*- and *z*-axis offsets, and thus enables robust angle estimation. Accordingly, pitch and yaw angle estimates are estimated by using the following models:

(4) $$\theta_{est}=a_{2}\frac{M_{23}}{M_{33}}+b_{2}$$

(5) $$\psi_{est}=a_{3}\frac{M_{32}}{M_{33}}+b_{3}$$

where $\theta_{est}$ and $\psi_{est}$ are estimated pitch and yaw angles, and $a_{1}$, $a_{2}$, $b_{2}$ and $b_{3}$ are the coefficients obtained from the calibration procedure. Another calibration procedure is conducted to estimate the *y*- and *z*-axis offsets. The calibration results show that $M_{21}$ and $M_{12}$ correlate linearly with *y*- and *z*-axis offsets. The *y*- and *z*-axis offsets are calculated as:

(6) $$y_{est}=a_{4}M_{21}+b_{4}$$

(7) $$z_{est}=a_{5}M_{12}+b_{5}$$

where $y_{est}$ and $z_{est}$ are estimated *y*- and *z*-axis offsets, and $a_{4}$, $a_{5}$, $b_{4}$, and $b_{5}$ are the calibration coefficients from the linear fit. However, the developed models for *y*- and *z*-axis are observed to change significantly with offsets in other degree-of-freedoms (DOF). For example, the *y*-axis model changes with yaw and the *z*-axis model changes with pitch. Therefore, the distance detection system is designed and calibrated to adapt to varying UUV pitch and yaw orientations with respect to that of the given light source. Here, the *y*- and *z*-axis estimates are updated based on the *x*-axis, pitch and yaw estimates based on 3-D vector analysis such that

(8) $$dx=x_{est}\left(k+1\right)-x_{est}\left(k\right)$$

(9) $$R=\frac{dx}{\cos\left(\theta_{est}\right)\cos\left(\psi_{est}\right)}$$

(10) $$dy=Rcos\left(\theta_{est}\right)\sin\left(\psi_{est}\right)$$

(11) $$dz=Rsin\left(\theta_{est}\right)$$

(12) $$y_{est}\left(k+1\right)=y_{est}\left(k\right)+dy$$

(13) $$z_{est}\left(k+1\right)=z_{est}\left(k\right)+dz$$

where dx is the distance travelled along the *x*-axis at the current time step, k. R is the magnitude of the 3-D vector between sequential UUV positions, and dy and dz are the calculated travel along the *y*- and *z*-axis, respectively. The pose detection algorithm is provided in Algorithm 1.

**Algorithm 1.** Pose EstimationObtain the image sampled on the detector arrayCompute $x_{est}$, $y_{est}$, $z_{est}$, $\theta_{est}$ and $\psi_{est}$Update $y_{est}$ based on $x_{est}$, $\theta_{est}$ and $\psi_{est}$Update $z_{est}$ based on $x_{est}$, $\theta_{est}$ and $\psi_{est}$Return $x_{est}$, $y_{est}$, $z_{est}$, $\theta_{est}$ and $\psi_{est}$

#### 2.2.2. Photodiode Response to Temperature Variations

To determine the temperature dependence, the photodiodes are immersed in a seven-liter water bath circulator at temperatures varying from 20 °C to 60 °C at 10 °C increments. A coherent light source (532 nm Z-Bolt SCUBA underwater dive laser) is used to provide a stable input to the photodiodes. Experimental results show that the output voltage from photodiodes decreases as the temperature increases. The temperature sensitivity is found to be 2 mV/°C as follows:

(14) $$V_{o}=-2\left(\frac{\mathrm{mV}}{{}^{\circ}C}\right)T\left({}^{\circ}C\right)+576\left(\mathrm{mV}\right)$$

where *T* is the water temperature, and $V_{o}$ is the voltage output. Based on the environment temperature, the voltage reading can be adjusted and the effect of the varying temperatures can be accounted for in the position and orientation detection algorithms.

#### 2.2.3. Hardware and System Cross-Talk

The major sources of hardware noise are: signal shot noise, $\sigma_{s}$; background shot noise, $\sigma_{b}$; dark-current shot noise, $\sigma_{dc}$; Johnson noise, $\sigma_{j}$; amplifier noise, $\sigma_{amp}$; and ADC-generated quantization noise, $\sigma_{q}$, as described in [28]. The hardware noise sources are assumed to be mutually independent and to be additive, uniform and unipolar [32]. Accordingly, the net noise is:

(15) $$\sigma_{n}=\sqrt{\sigma_{s}^{2}+\sigma_{b}^{2}+\sigma_{dc}^{2}+\sigma_{j}^{2}+\sigma_{amp}^{2}+\sigma_{q}^{2}}$$

In addition, cross-talk may occur from signal transmission in cables or in their connection to the reverse-bias circuitry. The net noise as measured from all the noise sources was in the range of 1 mV using 3.3 m SMA cables from the photodiodes to the reverse-bias circuitry and the serial communication losses. Therefore, it is concluded that the cable and circuit connection cross-talk in the system is not significant.

## 3. Results

### 3.1. Monte Carlo Simulations

The pose estimations (*x*-, *y*- and *z*-axis; pitch; and yaw) and TPU are obtained in the Monte Carlo simulations. The key steps include: (1) characterization of model uncertainty parameters; (2) generation of the uncertainty distribution for each hardware and environmental parameters (i.e., parameter ensemble); (3) simulation of the relative position and orientation estimations based on the parameter ensemble; and (4) evaluation of the position and orientation statistics for system TPU. Based on the calibration results, the uncertainty of four key parameters is characterized and shown in Table 1.

The hardware related uncertainties are determined as described in Section 2. Water column temperature variations of up to 3 °C and $K_{d}$ changes of up to 0.015 $m^{-1}$ are considered reasonable according to tank conditions. Uniform water conditions are assumed in the simulations (i.e., constant temperature and $K_{d}$ values in the water column). Environmental parameter variations (i.e., temperature and $K_{d}$) are assumed to have Gaussian distribution. The uncertainty parameters are used as input to the Monte Carlo simulation with a realization number of $N_{s}$ = 2000. A 5 × 5 image is created after each realization based on the relative pose between the light source and the detector array. Using the developed estimation models, the relative pose of the UUV to the light source is estimated and the pose statistics are extracted to determine system TPU.

The simulation conditions have the detector array mounted on the bow of the UUV. Two sets of reference trajectories are generated for the UUV to follow for pose estimation during the navigation (shown in Table 2):

1. The UUV undergoing diving motion (i.e., motion restricted to the xz-plane). The initial relative offsets between the light source and the optical detector array are Δx = 8.5 m, Δy = 0 m, Δz = 0 m, Δθ = 10°, and Δψ = 0°. The diving motion was conducted in clear ($K_{D}$ = 0.09 m^−1^) water conditions.
2. The UUV undergoing zigzag motion (i.e., diving and heading motion in 3-D space with initial offsets in the *y*- and *z*-axis). The initial relative offsets between the light source and the optical detector array are Δx = 8.5 m, Δy = 0.2 m, Δz = −0.1 m, Δθ = 0°, and Δψ = 10°. The zigzag motion simulation was conducted in clear ($K_{D}$ = 0.09 m^−1^) and turbid ($K_{D}$ = 0.2 m^−1^) water conditions.

In both trajectory simulations, the forward velocity, $u_{o}$, is assumed to be a constant 0.5 m/s. The UUV offset from the light beacon in x-direction ranges from 8.5 m to 4.5 m, which is designed to be consistent with the calibration experiments.

#### 3.1.1. Diving Motion

**Figure 6:**
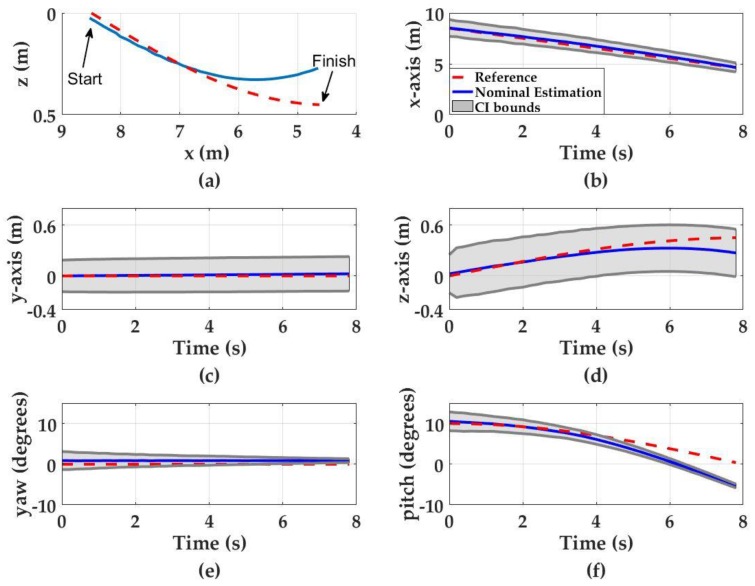
Monte Carlo simulation results when UUV undergoes diving motion. Red dashed curves denote the UUV motion, blue curves denote the nominal position estimations and the gray regions denote the 95% CI. (**a**) The UUV trajectory in diving motion; (**b**) *x*-axis estimations; (**c**) *y*-axis estimations; (**d**) *z*-axis estimations; (**e**) yaw estimations; and (**f**) pitch estimations.

The simulated navigation results of a UUV in diving motion are shown in Figure 6. The final nominal translational estimation errors (i.e., ensemble average of all the estimations for Ns = 2000) for the *x*-, *y*- and *z*-axis are 0.02 m, 0.02 m, and 0.18 m, respectively. The final nominal orientation estimation errors for pitch and yaw are 5° and 1°, respectively. The pose estimation Confidence Interval (CI) bounds (95%) along the *x*-axis and for yaw and pitch angles suggest a decreasing trend as the UUV approaches the light source. The CI bounds for the *x*-axis starts with 0.83 m at *t* = 0 s and reduces to ±0.43 m at *t* = 7.8 s. Similarly, the CI bounds for the pitch angle starts to within ±2° at *t* = 0 s then decreases to less than ±1° at *t* = 8 s. Although there is no yaw motion during the diving motion, hardware noise (described in the Section 2) degrades the input imagery and results with uncertainty estimations in yaw. Yaw uncertainty is approximately ±2° at the beginning of the motion and decreases to less than ±1° at the end of the motion. The decrease in pose estimation uncertainties is attributed to the increasing signal-to-noise ratio (SNR) as the UUV approaches the light source as demonstrated in Figure 5.

The CI bounds for both the *y*- and *z*-axis slightly increase as the UUV approaches the light source from ±0.18 m at the beginning of the motion to approximately ±0.2 m at the end of the motion for *y*-axis and ±0.22 m to ±0.28 m for *z*-axis. This is likely caused by the propagation of uncertainty in *x*-, pitch and yaw estimations, which are used to update the *y*- and *z*-axis estimations.

#### 3.1.2. Three-Dimensional Zigzag Motion in Clear Waters ($K_{D}$ = 0.09 m^−1^)

**Figure 7:**
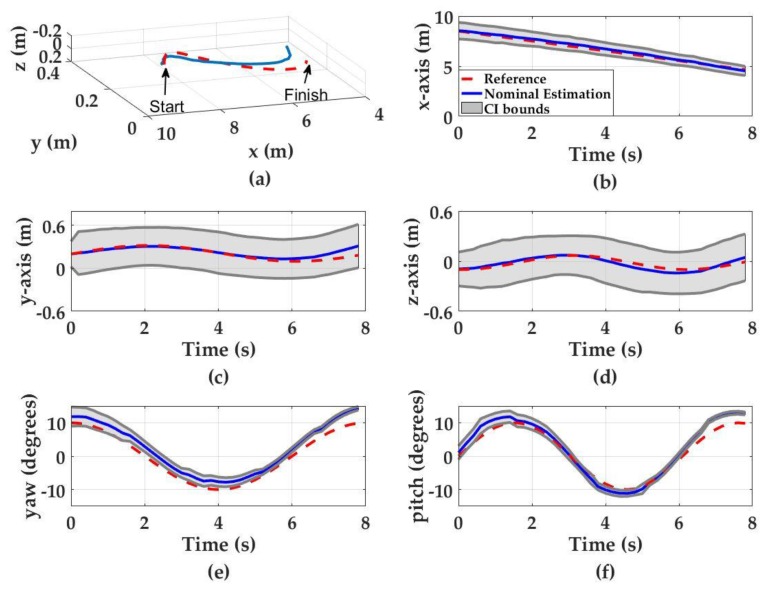
Monte Carlo simulation results for three-dimensional zigzag motion. Red dashed curves denote the UUV motion, blue curves denote the nominal position estimations and the gray regions denote the 95% confidence interval. (**a**) The UUV trajectory in zigzag motion (3D space); (**b**) *x*-axis estimations; (**c**) *y*-axis estimations; (**d**) *z*-axis estimations; (**e**) yaw estimations; and (**f**) pitch estimations.

The simulated navigation results of a UUV in 3-D zigzag motion are shown in Figure 7. The final nominal translational estimation errors (i.e., ensemble average of all the estimations for Ns = 2000) for the *x*-, *y*- and *z*-axis are 0.13 m, 0.13 m, and 0.05 m, respectively. The final nominal orientation estimation errors for pitch and yaw are 3° and 4°, respectively. The uncertainty values throughout the motion demonstrate a similar pattern to the diving motion. The estimation uncertainties associated with *x*-axis, pitch and yaw motions decrease as the UUV approaches the light source due to increasing SNR. The *x*-axis uncertainty starts from ±0.83 m at *t* = 0 s and decreases to ±0.46 m at *t* = 8 s. The pitch estimation uncertainty drops from ±2° to less than ±1°, whereas the yaw estimation uncertainty drops from ±3° to less than ±1°. The *y*- and *z*-axis estimation uncertainties increase from ±0.18 m at *t* = 0 s to ±0.3 m at *t* = 8 s for *y*-axis and from ±0.2 m to ±0.28 m for *z*-axis. Again, this increase is attributed to the propagation of uncertainty in *x*-, pitch and yaw estimations which are used to update the *y*- and *z*-axis estimations.

#### 3.1.3. Three-Dimensional Zigzag Motion in Turbid Waters $K_{D}$ = 0.2 m^−1^

**Figure 8:**
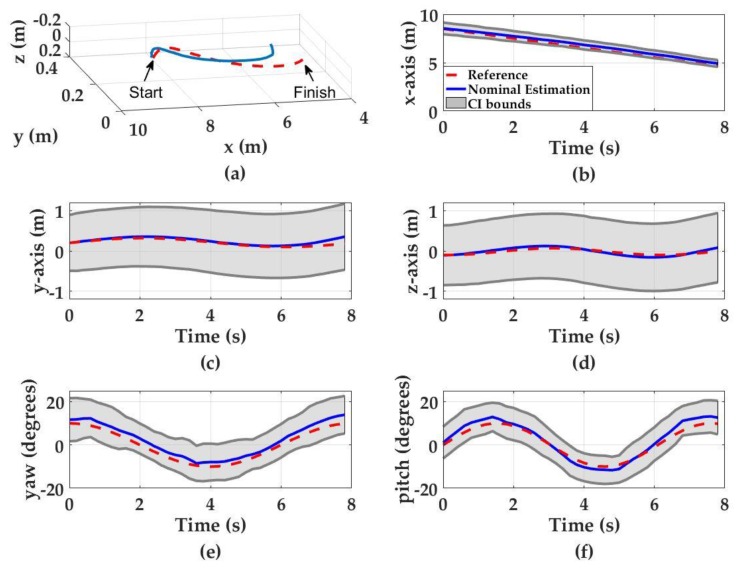
Monte Carlo simulation results for three-dimensional zigzag motion. Red dashed curves denote the UUV motion, blue curves denote the nominal position estimations and the gray regions denote the 95% confidence interval. (**a**) The UUV trajectory in zigzag motion (3D space); (**b**) *x*-axis estimations; (**c**) *y*-axis estimations; (**d**) *z*-axis estimations; (**e**) yaw estimations; and (**f**) pitch estimations.

The performance of the pose detection system is also tested in waters with higher turbidities. Monte Carlo simulations are conducted to represent Portsmouth Harbor, NH conditions as a potential deployment site ($K_{D}$ = 0.2 m^−1^) [33,34]. The zigzag motion demonstrated in Section 3.1.2 is used to compare the performance of the system under the same configuration. The simulation results in turbid water conditions demonstrate that the final nominal errors do not change significantly when compared to simulations conducted in clear waters ($K_{D}$ = 0.09 m^−1^) (Figure 8). The final nominal estimation errors for the *x*-, *y*- and *z*-axis are 0.26 m, 0.17 m and 0.01 m, respectively. These results indicate 0.13 m increase in *x*-axis, 0.04 m increase in *y*-axis, and 0.04 m decrease in *z*-axis. The final nominal orientation estimation errors for pitch and yaw are 3° and 4°, respectively, and are similar to the simulation results conducted in clear waters. However, higher turbidity results in higher pose detection uncertainties, especially for the *y*-axis, *z*-axis, pitch and yaw estimations. The simulation results show that final pose detection uncertainties are ±0.36 m, ±0.8 m and ±0.86 for *x*-, *y*- and *z*-axis, respectively, indicating a 0.1 m decrease in *x*-axis, 0.5 m increase in *y*-axis and 0.6 m increase in *z*-axis uncertainty compared the results obtained in less turbid water conditions. Similarly, the final pitch and yaw detection uncertainties are ±8° and ±9° for pitch and yaw, respectively, which indicate 7° pitch and 6° yaw increase in uncertainty, compared to the simulation results obtained in less turbid water conditions. The increase in pose detection uncertainty in turbid waters is attributed to the light attenuation and diffusion of the light beam due to multi-path scattering.

### 3.2. Empirical Measurements

**Figure 9:**
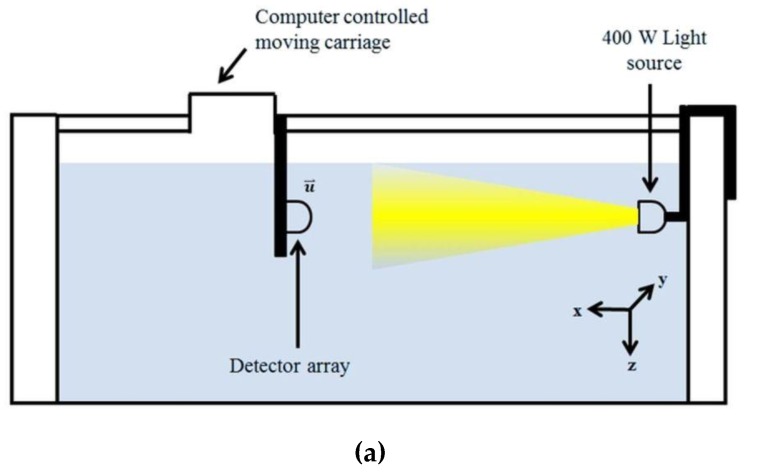

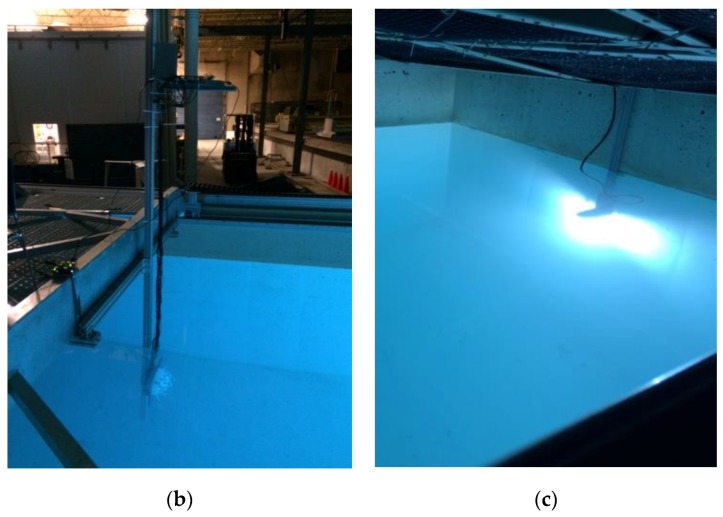
(**a**) Schematic diagram of the experimental setup in the wave and tow tank; (**b**) image of the detector array submerged into the water on the moving carriage platform; and (**c**) image of the light source mounted on the wall of the tank.

The detection performance of the optical detector array system is also evaluated through empirical measurements at the UNH Ocean Engineering facilities. The metal halide light source was used as a guiding beacon and placed on the wall of the wave/tow tank (Figure 9). The detector array is mounted on a tow carriage with the ability to vary translational offsets, (i.e., varying *x*-, *y*- and *z*-axis positions to simulate UUV trajectories).

Two sets of dynamic wave/tow tank tests are conducted to evaluate the detector array’s pose detection capability. In the first test, there is no *yz*-offset between the light source and the detector array (i.e., the light source and the detector array positioned at a center-to-center distance from each other) with an 8.5 m *x*-axis separation (i.e., *x* = 8.5 m, *y* = 0 m and *z* = 0 m). In the second test, the detector array offset relative to the light source is maintained within a maximum offset of *x* = 8.5 m, *y* = 0.6 m and *z* = 0.8 m. In both tests, the detector array initial position is *x* = 8.5 m away from the light source and then approaches the light source with a velocity of 0.5 m/s. After the detector array reaches its final position (*x* = 4.5 m away from the light source), the detector array is kept stationary to observe the detection performance during UUV station keeping.

#### 3.2.1. Test 1 Results

**Figure 10:**
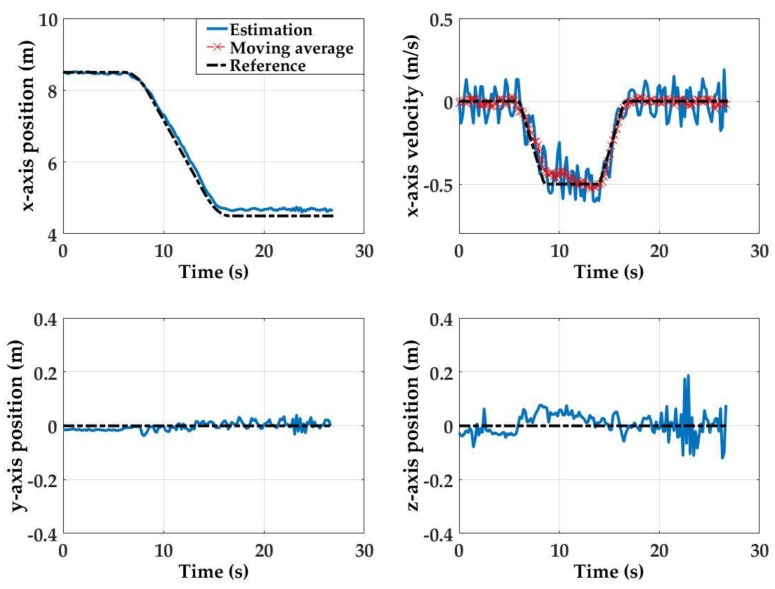
Empirical results for position estimations for Test 1: (**a**) reference and *x*-axis position estimate; (**b**) velocity reference and raw and smoothed velocity estimates; (**c**) raw and smoothed *y*-axis position estimates; and (**d**) raw and smoothed *z*-axis position estimates.

For Test 1, the initial state is such that *x* = 8.5 m, *y* = 0 m, and *z* = 0 m, and the final state at *x* = 4.5 m, *y* = 0, and *z* = 0 m. This first set of empirical results shows that the detector array estimation performance is highly accurate throughout the detector array motion (see Figure 10). At the initial state (*x* = 8.5 m away from the light source), position estimation errors for all axis are within 0.02 m. Here, because the estimation results for *y*-axis, *z*-axis and *x*-axis velocity estimates are noisy, they are smoothed with a moving average window (bin size: 10 samples).

The steady state estimation errors are 0.1 m, 0.0 m and 0.08 m for the *x*-, *y*- and *z*-axis, respectively. The *x*-axis velocity estimations are also within 0.2 m/s throughout the course of the motion with a final error of 0.07 m/s. Measurement noise observed in the *y*-axis, *z*-axis and forward velocity estimations can be attributed to the combination of hardware noise, mechanical vibrations along the tow carriage platform path, and environmental factors (e.g., non-uniform water conditions). Noise is most prevalent along the *z*-axis after the detector array has reached the final stationary state. The observed noise can be attributed to the pitching of the detector platform even after the forward motion of the tow carriage is stopped.

#### 3.2.2. Test 2 Results

**Figure 11:**
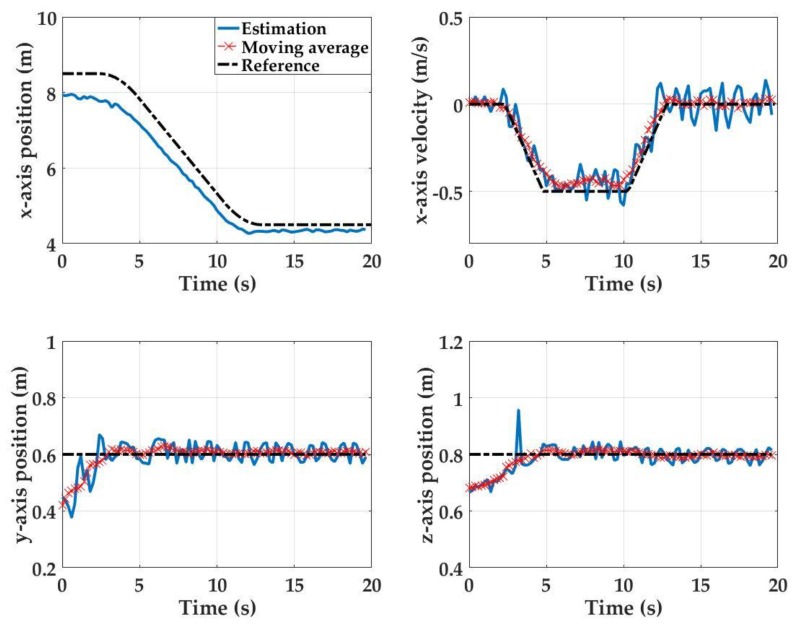
Empirical results for position estimations for Test 2: (**a**) reference and *x*-axis position estimate; (**b**) velocity reference and raw and smoothed velocity estimates; (**c**) raw and smoothed *y*-axis position estimates; and (**d**) raw and smoothed *z*-axis position estimates.

For Test 2, the initial state is at *x* = 8.5 m, *y* = 0.6 m, and *z* = 0.8 m, and the final state is at *x* = 4.5 m, *y* = 0.6, and *z* = 0.8 m. At the beginning of Test 2, when the platform is stationary at its initial starting position, position estimation errors of 0.5 m, 0.15 m and 0.15 m are observed in the *x*-, *y*- and *z*-directions, respectively (Figure 11). However, as the detector array approaches the light source, these errors reduce and finally reach to 0.1 m, 0.01 m and 0.01 m at the final stationary state. This phenomenon is also attributed to an increasing SNR as the UUV approaches to the light source. Maximum *x*- and *z*-axis estimation errors of 0.8 m and 0.2 m, respectively, are observed when the system transitions from being stationary to becoming dynamic at *t* = 3 s. The maximum *y*-axis initial position estimation error is 0.2 m. The detection system is able to track the carriage velocity with a maximum velocity estimate error of 0.14 m/s error during the platform motion.

## 4. Discussion

The evaluation of the pose detection results showed that the developed system can provide satisfactory pose detection performance. The errors obtained for all directional motions (*x*-, *y*- and *z*-axis; pitch; and yaw) were within the required positioning criteria. Monte Carlo simulation results showed that the pose estimation parameters demonstrated in this study were good indicators of pose. Specifically, the ratio of the image moment matrix indices used for pitch and yaw detection (i.e., $\frac{M_{23}}{M_{33}}$ for pitch and $\frac{M_{32}}{M_{33}}$ for yaw) were invariant to changes in offsets in all axial directions (Figure 5). As a result, single models for pitch and yaw estimations were developed that were valid throughout the UUV navigation. Similarly, the logarithmic model based on the photodiode intensity was also a good indicator of *x*-axis estimates. The pose estimation uncertainties in *x*-axis, pitch and yaw directions significantly decreased as the distance between the detector array and the light source decreased. At closer ranges, the pose detection models performed better, as a result of the increasing SNR of the image sampled on the detector array. This finding was also verified by the dynamic empirical experiments. The initial estimation errors in the *x*-, *y*- and *z*-axis observed at the beginning of the motion ($\Delta x_{i}$ = 0.5 m, $\Delta y_{i}$ = 0.15 m and $\Delta z_{i}$ = 0.15 m) decreased significantly at the end of the motion ($\Delta x_{f}$ = 0.1 m, $\Delta y_{f}$ = 0.01 m and $\Delta z_{f}$ = 0.01 m). In this study, the positioning requirements were based on hovering and docking applications [7,9,25]. However, it should be mentioned that the positioning requirements vary with respect to the size and design configuration. For example, in cone type docking station used in [9], error tolerances in *y*- and *z*-axis were set as ±1 m and yaw error tolerance was set to be within ±45°. Another example is the pole type docking stations in which the UUV latches onto a pole within 1 m of the pole [35]. This type of design makes the *x*-axis positioning requirements more flexible but require tighter tolerances in orientation.

The main challenge in the pose detection algorithms is to estimate five DOF motion from a single image. This challenge manifests itself in coplanar motion. For example, even with zero *y*- or *z*-axis offsets, yaw angle motion offsets cause erroneous *y*-axis detection whereas pitch angle motion offsets cause erroneous *z*-axis estimations. Thus, decoupling specific motions is not trivial (i.e., differentiating *y*-axis displacement from yaw motion and *z*-axis displacement from pitch motion). To overcome this challenge, the estimated *x*-axis, pitch and yaw motions were used in 3-D vector geometry to update the *y*-axis and *z*-axis positions as described in Section 2. As observed from the Monte Carlo simulations, the propagation of uncertainties in *x*-axis, pitch and yaw angle estimations increased the uncertainty in *y*- and *z*-axis estimations. As the increasing pose estimation uncertainties in *y*- and *z*-axis may adversely affect the UUV positioning performance, it is recommended to further improve the pose algorithms by fusing the data from other sensors that are located on the UUV (e.g., Inertial Measurement Unit and Doppler Velocity Log) with the output from the optical detector array.

**Figure 12:**
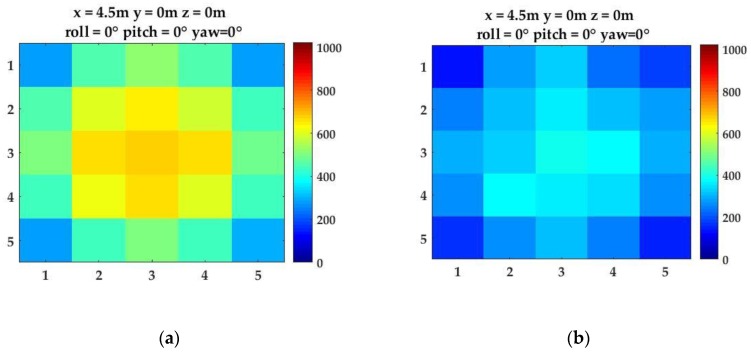
The sampled light field in the simulator in two different water clarity conditions at the same distance to the light source: (**a**) image sampled in relatively clear waters with $K_{D}$ = 0.09 m^−1^; and (**b**) image sampled in turbid waters with $K_{D}$ = 0.2 m^−1^.

The empirical measurements and Monte Carlo simulations identified a variety of factors that contribute to the accuracy of the pose detection demonstrated in this paper. First, the developed system is a proof-of-concept detector array constructed with cost efficient commercial off-the-shelf optical components and ADCs. The pose detection performance could be improved by using ADCs with higher resolution, e.g., 16-bit ADC, which could increase the radiometric resolution sampled on the detector array. In addition, using a green light source at 532 nm and a photodetector with peak sensitivity approximately at 532 nm could increase both effective range and the pose detection performance. Second, water clarity plays a critical role in the pose detection performance. The study results showed the detector array performance in the UNH wave/tow tank conditions with average $K_{D}$ = 0.09 m^−1^. By using the developed simulator, it is possible to predict the performance of the detector array for different diffuse attenuation coefficients using Beer’s law [27,36]. The pose detection performance of the optical detector array systems was also evaluated by simulating turbid water conditions in Portsmouth Harbor, NH with an average $K_{D}$ = 0.2 m^−1^. The results showed that the pose estimation uncertainties significantly increased in turbid waters. In addition to the light energy attenuation, the turbid water conditions cause multi-path scattering that diffuse the light beam and result in a uniform distribution over the detector array. The comparison of images obtained in clear and turbid water conditions is demonstrated in Figure 12.

In Figure 12a, in clear waters, the Gaussian light field can be observed on the detector array. As the turbidity increases, the light beam is attenuated and diffused. Thus, the sampled light field on the detector array in turbid waters deviates from Gaussian beam characteristics (Figure 12b). As a result, the position detection algorithms developed for the Gaussian beam assumption result in significantly higher pose estimation uncertainty in turbid waters than in clear waters (Section 3.1.3). In addition, in field experiments, Beer’s law assumption may not be sufficient to model the underwater light field conditions [37]. These results demonstrate the importance of conducting pose calibration of the proposed system in field experiments for enhanced performance.

Other potential factors that affected the performance of the pose detection system includes additional environmental factors, such as inhomogeneous water clarity, currents and biofouling. Sediment plumes occurring in water can create water clarity inhomogeneity and therefore have the potential to significantly reduce the visibility conditions in the water. Water waves and currents also play a detrimental role during UUV positioning, e.g., docking. These forces cause both platforms (i.e., light source and UUV) to drift out of position. To minimize these effects, the light beacon should be mechanically designed to withstand these forces. In terms of a UUV, the currents can be measured onboard with a velocity sensor [9]. These measurements can then be used to compensate for the current-induced disturbance forces in the UUV control system (e.g., via automatic feedforward control). Copper components should be included in the design of the detector array and light source to prevent biofouling to cause issues in long underwater deployments [12].

## 5. Conclusions

The goal of this study was to evaluate the performance of a proposed optical detector array unit for relative positioning of a UUV with respect to a light source. Monte Carlo simulations and empirical measurement results with a 5 × 5 photodetector array showed that the maximum pose estimation errors were within 0.13 m, 0.13 m, and 0.18 m for the *x*-, *y*- and *z*-directions, respectively; within 5° for pitch and yaw; and within 0.2 m/s for *x*-axis velocity estimations. The TPU of the pose detection system was also evaluated by taking into account the variation in hardware and environmental parameters that could degrade the system performance. The Monte Carlo simulation results showed that the pose estimation uncertainty (2σ) of the detector array at noise levels of 1% of the maximum photodiode intensity were less than ±0.46 m in the *x*-direction, ±0.3 m for the *y*-direction and the *z*-direction, and less than ±1° for pitch and yaw motion. These results support the potential use of a detector array for UUV positioning applications in acoustically noisy environments, such as in Portsmouth Harbor, NH.

## Figures and Tables

**Table 1:** Uncertainty parameters used in the Monte Carlo simulations.

Parameter	Standard Deviation (1σ)
Water column temperature variation	3 °C
Net electronic noise (max)	50 mV
$K_{d}$	0.015 $m^{-1}$

**Table 2:** UUV Reference trajectories used in the Monte Carlo simulations.

Case I UUV Diving Motion					Case II UUV Zigzag Motion			
Time (s)	*t* = 0	*t* = 2.6	*t* = 5.2	*t* = 8	*t* = 0	*t* = 2.6	*t* = 5.2	*t* = 8
*x* (m)	8.5	7.22	5.93	4.53	8.5	7.22	5.94	4.56
*y* (m)	0	0	0	0	0.2	0.31	0.12	0.2
*z* (m)	0	0.22	0.38	0.45	−0.1	0.06	−0.06	0
pitch (˚)	10	8.7	5.2	0	0	4.5	−8	9.2
yaw (˚)	0	0	0	0	10	−4.5	−5.9	10
$u_{o}$ (m/s)	0.5	0.5	0.5	0.5	0.5	0.5	0.5	0.5
